# Oxidant/Antioxidant Status of Breast Cancer Patients in Pre- and Post-Operative Periods

**DOI:** 10.3390/medicina56020070

**Published:** 2020-02-11

**Authors:** Janina Didžiapetrienė, Birutė Kazbarienė, Renatas Tikuišis, Audrius Dulskas, Daiva Dabkevičienė, Vaida Lukosevičienė, Eglė Kontrimavičiūtė, Kęstutis Sužiedėlis, Valerijus Ostapenko

**Affiliations:** 1 National Cancer Institute, LT-08660 Vilnius, Lithuania; janina.didziapetriene@nvi.lt (J.D.); birute.kazbariene@nvi.lt (B.K.); renatas.tikuisis@nvi.lt (R.T.); daiva.dabkeviciene@nvi.lt (D.D.); vaida.lukoseviciene@nvi.lt (V.L.); kestutis.suziedelis@nvi.lt (K.S.); valerijus.ostapenko@nvi.lt (V.O.); 2 Clinic of Internal Diseases, Family Medicine and Oncology, Faculty of Medicine, Vilnius University, LT-01513 Vilnius, Lithuania; 3 Clinic of Anesthesiology and Intensive Care, Faculty of Medicine, Vilnius University, LT-01513 Vilnius, Lithuania; egle.kontrimaviciute@santa.lt; 4 Center of Anesthesiology, Intensive Care and Pain Management, University Hospital Santaros Clinics, LT-08661 Vilnius, Lithuania

**Keywords:** breast cancer, surgery, oxidant/antioxidant status, oxidative stress, malondialdehyde

## Abstract

*Background and Objectives:* The purpose of this study is to evaluate the level of oxidative stress before and after breast cancer surgery. *Materials and Methods:* Malondialdehyde (MDA) level was tested using a thiobarbituric acid (TBA) assay based on the release of a color complex due to TBA reaction with MDA. The glutathione S-transferase (GST) activity was evaluated by enzymatic conjugation of reduced glutathione (GSH) with 1-chloro-2,4-dinitrobenzene. The level of total glutathione (reduced GSH and oxidized GSSG) was detected using a recycling system by 5,5-dithiobis(2-nitrobenzoic acid). The levels of the indices were determined in the serum of 52 patients before surgery, two hours and five days after surgery, and in 42 healthy women. *Results:* In the patients over 50 years old the level of MDA was higher after surgery in comparison with before surgery, and GST activity was lower in comparison with the control. The GSH + GSSG level in both ages groups after surgery was lower than in the control. Significant differences of MDA level were detected in patients with stage III after surgery compared to the control. The level of GSH + GSSG was significantly lower in the patients with I–III stages compared to the control. *Conclusion:* The most expressed changes demonstrate the significance of MDA as a marker to evaluate oxidative stress in breast cancer patients. The degree of oxidative stress depends on the patient’s age and stage of disease. (1) Malondialdehyde can be used as an oxidative stress marker; (2) A higher stage of the disease and older age correspond to a higher rise of malondialdehyde, suggesting more intensive oxidative stress.

## 1. Introduction

Breast cancer is one of the most relevant of women’s health issues worldwide. Within the category of causes of death, breast cancer is the leading cause of death from malignant tumors in women [1]. Known risk factors can explain the causes for the development of breast tumors only in a small proportion of patients [2]. Breast cancer treatment is comprised of surgical intervention with chemotherapy, radiation and hormonal therapy, in either the neoadjuvant and/or adjuvant setting [3,4,5].

Growing experimental and clinical evidence reveals that reactive oxygen species (ROS) and their reactive derivatives are involved in carcinogenesis and the progression of breast cancer [6]. Trauma caused by surgery is associated with excessive generation of ROS and/or a decreased antioxidants level in cells. This imbalance between ROS and the impaired antioxidant defense results in oxidative stress [7].

Oxidative stress is assessed by detection of lipid, protein, or DNA oxidation markers. The intensity of the lipid peroxidation is determined by measurement of the final lipid peroxidation products. The main lipid peroxidation final product is malondialdehyde (MDA). It is an indicator of oxidative stress. Glutathione and glutathione-dependent enzymes (such as glutathione peroxidase, glutathione S-transferase (GST) and glutathione reductase) are involved in cell defense against ROS [8,9,10,11]. Many reports indicate the development of oxidative stress in cancer patients [12,13,14,15]. However, there is not much research regarding the level of oxidative stress during the treatment. Probasheela et al. [16,17] revealed differences in the lipid peroxidation and antioxidant status in the blood of cancer patients before and after surgery; while El-Hefny et al. [18] found no significant differences in lipid peroxidation and antioxidant status between pre- and post-operative patients, as well as after chemotherapy. It is important to note that the level of oxidative stress depends on many factors, including the patient’s status (age, body weight, hormone balance) and cancer characteristics.

Therefore, the aim of this study is to evaluate the level of oxidative stress before and after breast cancer surgery concerning the age of patients and the stage of disease.

## 2. Materials and Methods

Fifty-two women with breast cancer (ductal carcinoma in 46, invasive lobular carcinoma in three and other—two) and 42 healthy women (control) were enrolled in the study. The study was conducted at the National Cancer Institute.

The Regional (Vilnius) Bioethics Committee approved the study (2009-12-02 No. 158200-12-101-34). All methods were performed in accordance with the relevant guidelines and regulations. All the participants were informed, and written informed consent was obtained prior to entry into the study. Table 1 shows the general characteristics of patients included in the study. Exclusion criteria were clinical or pathologic evidence of cancer at any other site, and liver dysfunction, diabetes mellitus, heart failure, renal failure, oral contraceptives or antioxidants supplementation.

Patients with breast cancer were classified using the American Joint Committee on Cancer (AJCC) tumor/node/metastasis (TNM) classification and staging system. Patients were operated under general anesthesia with laryngeal mask insertion. The surgical procedures for the breast included breast-conserving surgery. The control group consisted of 42 healthy women. Fasting blood samples were collected in the control group and as well as in the patients group the day before surgery, and two hours and five days after surgery. Serum samples were stored at −70 °C.

Subgroup analysis was planned in order to analyze patients in accordance to cancer stage and age.

### 2.1. Analysis of MDA, Total Glutathione (GSH + GSSG) and GST

The level of the lipid peroxidation product MDA (nmol/mL) was determined spectrophotometrically (NanoDrop 2000c, Thermo Scientific, Vilnius, Lithuania was used). MDA was tested using a thiobarbituric acid (TBA) assay based on the release of a color complex due to a TBA reaction with MDA [19]. The GST activity (µmol/mL/min) was evaluated by enzymatic conjugation of GSH with 1-chloro-2,4-dinitrobenzene (CTNB) [20]. The level of functional antioxidant total glutathione (μmol/mL) both in reduced and oxidized form (GSH + GSSG) was detected using a recycling system using 5,5-dithiobis(2-nitrobenzoic acid) (DTNB) [21].

### 2.2. Reagents

Reagents used were obtained from commercial producers: DTNB (D8130; Merck, Darmstadt, Germany), Glutathione reductase (G3664, Merck), GSH (G4251, Merck), HCl (T134, Carl ROTH, Karlsruhe, Germany), NADPH (N1630, Merck), TBA (T5500, Merck), TCA (8789, Carl ROTH) and CTNB (237329, Merck).

### 2.3. Statistical Analysis

Means ± standard deviation (M ± SD) are reported. The non-parametric Friedman test was used to examine possible differences in the measurements among the three phases. As a post hoc test for Friedman’s test, the Wilcoxon signed-rank test was conducted, with corrections of the *p*-value according to the Bonferroni. The Mann–Whitney test was used to compare outcomes between groups. *p*-values were considered significant if <0.05, and in post-hoc comparisons if <0.017 (Bonferroni correction). Statistical analysis was performed using SPSS 21.0 and STATISTICA 10.0 (StatSoft, Tulsa, OK, USA).

## 3. Results

Table 2 shows the mean serum levels of MDA, GSH + GSSG and GST activity in breast cancer patients before surgery, two hours and five days after the surgery, and in the control subjects.

**Figure 1:**
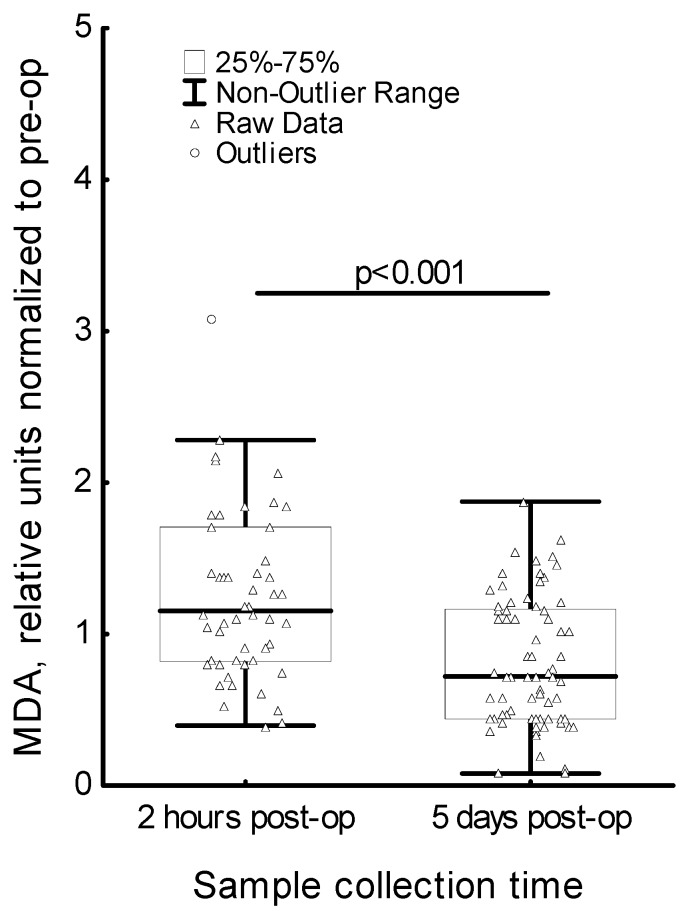
Serum level of malondialdehyde (MDA) in breast cancer patients in regard to sample collection time. MDA relative units of two hours post-op and five days post-op denote the MDA level ratio: two hours post-op/pre-op and five days post-op/pre-op, respectively.

**Figure 2:**
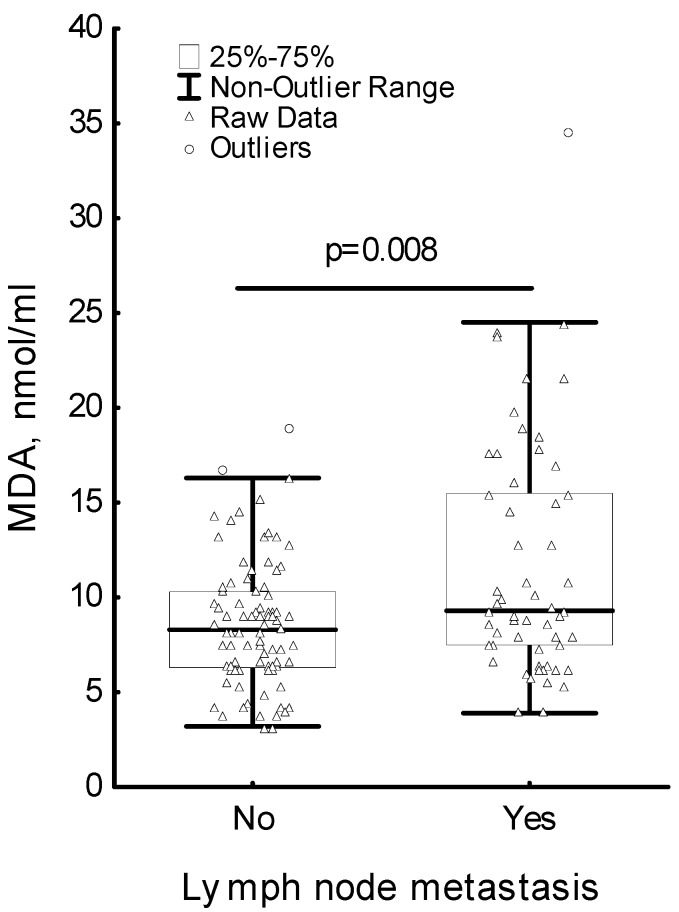
Serum level of malondialdehyde in breast cancer patients in regard to lymph node metastasis.

The MDA level statistically significantly increased two hours after surgery compared to the MDA level before surgery; and five days after surgery was it significantly lower in comparison to the level two hours after surgery. To support these findings, we evaluated changes in MDA level after surgery for each patient. The ratio of the MDA levels of each patient after surgery (post-op) and pre-operative (pre-op) MDA levels was included to the analysis. The relative amount of MDA at two hours post-op was found to be significantly higher than the relative amount of MDA at five days post-op (*p* < 0.001) (Figure 1). The analysis of the changes in the MDA level, GST activity and GSH + GSSG level in regard to lymph node metastasis has shown significant differences only in the MDA level (Figure 2). Patients positive for lymph node metastasis had a significantly higher MDA level than patients negative for lymph node metastasis (*p* = 0.008). The MDA level did not reach the MDA level in the control group. There were no statistically significant differences in the GST activity as well as in the GSH + GSSG levels before surgery, as well as two hours or five days after the surgery. At the same time, the GST activity and GSH + GSSG level in breast cancer patients was lower compared to their levels in the control group in both cases.

The analysis of the changes in the MDA level, GST activity and GSH + GSSG level in regard to the patient’s age has shown the most significant differences in the MDA level (Table 3). In breast cancer patients under 50 years of age, statistically significant differences were not observed. In breast cancer patients over 50 years of age, the MDA level two hours after surgery increased in comparison to its level before surgery, and decreased five days after surgery in comparison to its level two hours after surgery. However, the MDA level did not reach the MDA level in the healthy control group.

In both groups of patients, there were no statistically significant differences between the GST activity before surgery, and two hours and five days after surgery.

The GSH + GSSG level was similar in both groups of breast cancer patients under and over 50 years of age, and five days after surgery and it was lower in comparison with the GSH + GSSG level in the control group. There were no significant differences between the GSH + GSSG level before surgery, and two hours and five days after surgery.

The analysis of the changes in the level of MDA, in the activity of GST and level of GSH + GSSG revealed differences in regard to the stage of disease (Table 4).

The most expressed changes were established in respect to the MDA level. The data obtained illustrate the significance of MDA as an indicator of oxidative stress.

## 4. Discussion

Excessive oxidative stress arises because of an enormous production of free radicals and the decreased capacity of the non-enzymatic and enzymatic antioxidant defense system to counteract an oxidative stress.

Surgical trauma results in increased ROS production due to mechanical damage and hypoxia, as well as the decrease of antioxidant protection due to the increase of ROS scavengers, and leads to adverse effects caused by oxidative stress on the host organism. Post-operative oxidative stress caused by surgical interventions is well documented in different pathologies, including cancer [7,22,23,24,25]. However, there is little data regarding oxidative damage due to surgical treatment of breast cancer patients.

Many reports have demonstrated that MDA is an indicator of oxidative damage, and its level in cancer patients is higher in comparison with control subjects [13,26]. The results regarding the changes in the MDA level in breast cancer patients before and after surgery are contradictory. According to the results obtained by Prabasheela and co-authors [16], the lipid peroxidation level—which was estimated by measuring the thiobarbituric acid reactive substances (TBARS)—and the conjugated dienes and hydro peroxides in the serum of breast cancer patients after surgery was lower than the level before surgery. El-Hefny and co-authors [18] found no significant differences in the MDA level in breast cancer patients prior to the surgery compared to three weeks after surgery, while Szychta and co-authors [27] estimated that surgery for breast cancer induces an increase in oxidative damage to membrane lipids, when MDA + 4-MDA was measured in the early post-operative period (24 hours after surgery). The different results obtained by the above-mentioned authors, it seems, depend on many factors, including time of measurements, age of patients, and stage of disease. According to the present study, the changes in the MDA level depended on the age of patients and the stage of disease. The most significant changes in the MDA level were found in breast cancer patients over 50 years of age, when the homeostasis of the organism is more restored. The highest MDA level was observed in breast cancer patients of stage III, due, it seems, to a possibility of the accumulation of free radicals, which manifests in significantly higher lipid peroxidation. Therefore, our findings are in agreement with most of the studies that reported that the MDA level in cancer patients, including breast cancer patients, is elevated compared to the control subjects, and that its level increases in the early post-operative period.

The results regarding the glutathione system status in cancer patients are contradicting. Some authors [28] have found a decrease of antioxidant levels of glutathione as well as glutathione-dependent enzymes in the blood of oral cancer patients; whereas others [29,30] have reported an increase in the levels in colorectal and breast cancer patients compared to normal individuals. The different results obtained may depend on the type of tumor, the pathological characteristics of tumors, and the clinical characteristics of the patients. According to Scibior and co-authors [25], the glutathione level was significantly lower in patients with gastric cancer and higher in patients with colorectal cancer liver metastases, and no differences were observed in liver cancer and colorectal patients compared to control subjects. The authors have also found that the changes in glutathione-dependent enzymes activities are associated with different types of gastrointestinal tract tumors.

Lower serum levels of GSH and higher activity of GST were observed in post-operative breast cancer patients as compared to the levels in control subjects [31]. It is important to note that GST expression in breast cancer patients was not significantly correlated with the hormone status of the tumors. However, GST expression showed a significantly positive correlation with the lower histological grade/C-erb-B2 negative breast carcinoma phenotype [32].

Our study aimed to evaluate the level of GSH + GSSG and activity of GST before and after surgery concerning the age of patients and stage of disease. Our findings are in agreement with previously reported studies [31]—that the GSH + GSSG level in breast cancer patients is lower, but, in contradiction, the GST level was also lower when compared to the control subjects. We did not find differences between GSH + GSSG or GST levels before surgery, after surgery, and five days after surgery, while according to Prabasheela and Baskaran [17], the activity of glutathione-dependent enzymes was found to be decreased after surgery. The different results can be influenced by the time of the measurement of the glutathione-dependent enzymes levels after surgery.

Our results, together with data reported in the literature, confirmed alterations in lipid peroxidation in breast cancer patients. These alterations lead to oxidative stress, and the main indicator of oxidative stress is the MDA level. On the other hand, our findings showed differences in MDA and GSH levels and GST activity in regards to the age of patients, stage of disease and surgical intervention. As there is an association between the level of oxidative stress and cancer progression, further studies are needed to evaluate the obtained changes of parameters as reflecting oxidative stress in breast cancer patients in association with response to treatment, especially taking into account the values of parameters as regards lipid peroxidation and the antioxidant system status.

## 5. Conclusions

The most significant changes are established in respect to the MDA level, and this demonstrates the significance of MDA as a marker to evaluate the degree of oxidative stress in breast cancer patients.

Further studies are in progress to evaluate the level of these parameters in relationship to the survival of breast cancer patients.

## Figures and Tables

**Table 1:** Clinicopathologic characteristics of breast cancer patients (*n* = 52).

Parameters	Number of Cases (*n*)
Age	
<50	34
>50	18
Histologic subtype	
Ductal carcinoma	46
Invasive lobular carcinoma	3
Other	3
TNM stages	
0	3
I	19
II	22
III	8
Lymph node metastasis	
Yes	18
No	34
Tumor grade	
G_x_	6
G_1_	8
G_2_	23
G_3_	15
Type of surgery	
Breast conserving surgery	52
Body mass index under 50 years of age	
<18.5 18.5–24.9 25–29.9 30–34.9 35–40 Body mass index over 50 years of age	– 15 12 5 2
<18.5 18.5–24.9 25–29.9 30–34.9 35–40	– 4 9 4 1

**Table 2:** Serum level of malondialdehyde, activity of glutathione S-transferase and level of total glutathione in breast cancer patients and in the control subjects.

Parameters	Before Surgery	Two Hours after Surgery	Five Days after Surgery	Control
MDA, nmol/mL	11.29 ± 7.69 *	13.93 ± 8.35 *	9.83 ± 3.15 ^●^	6.73 ± 2.66 ^●^
GST, µmol/mL/min	0.36 ± 0.16	0.37 ± 0.16	0.37 ± 0.16 ^●^	0.48 ± 0.13 ^●^
GSH + GSSG, µmol/mL	0.47 ± 0.19	0.46 ± 0.3	0.48 ± 0.21 ^●^	0.79 ± 0.28 ^●^

GSH + GSSG = total glutathione; GST = glutathione S-transferase; MDA = malondialdehyde. * *p* < 0.017; ^●^ *p* < 0.05.

**Table 3:** Serum level of malondialdehyde, activity of glutathione S-transferase and level of total glutathione in breast cancer patients and control group in regards to age.

**Under 50 Years of Age**						
**Parameters**	***n***	**Before Surgery**	**Two Hours after Surgery**	**Five Days after Surgery**	***n***	**Control**
MDA, nmol/mL	34	10.04 ± 5.2	11.68 ± 8.39 *	8.94 ± 4.24 *	20	6.06 ± 1.72
GST, µmol/mL/min	34	0.33 ± 0.16	0.34 ± 0.17	0.33 ± 0.16	20	0.41 ± 0.19
GSH + GSSG, µmol/mL	34	0.51 ± 0.21	0.46 ± 0.14	0.49 ± 0.24 ^●^	20	0.98 ± 0.13 ^●^
**Over 50 Years of Age**						
**Parameters**	**n**	**Before Surgery**	**Two Hours after Surgery**	**Five Days after Surgery**	**n**	**Control**
MDA, nmol/mL	18	13.67 ± 6.76 *	18.18 ± 10.76 *	11.46 ± 6.30 ^●^	22	7.40 ± 1.83 ^●^
GST, µmol/mL/min	18	0.43 ± 0.13	0.44 ± 0.14	0.39 ± 0.17 ^●^	22	0.55 ± 0.16 ^●^
GSH + GSSG, µmol/mL	18	0.41 ± 0.13	0.41 ± 0.11	0.47 ± 0.15 ^●^	22	0.60 ± 0.17 ^●^

GSH + GSSG = total glutathione; GST = glutathione S-transferase; MDA = malondialdehyde; *n* = number of patients.* *p* <0.017; ^●^ *p* <0.05.

**Table 4:** Serum level of malondialdehyde, activity of glutathione S-transferase and level of total glutathione in breast cancer patients and the control group in regard to the stage of disease.

**Stage I**					
**Parameters**	**n**	**Before Surgery**	**Two Hours after Surgery**	**Five Days after Surgery**	**Control**
MDA, nmol/mL	19	11.99 ± 9.08 *	16.04 ± 10.22 *	10.52 ± 6.11 ^●^	6.73 ± 2.66 ^●^
GST, µmol/mL/min	19	0.38 ± 0.15	0.39 ± 0.14	0.39 ± 0.13	0.48 ± 0.23
GSH + GSSG, µmol/mL	19	0.48 ± 0.20	0.48 ± 0.09	0.51 ± 0.28 ^●^	0.79 ± 0.28 ^●^
**Stage II**					
MDA, nmol/mL	22	8.91 ± 3.47	10.30 ± 4.41	8.23 ± 3.27	6.73 ± 2.66
GST, µmol/mL/min	22	0.36 ± 0.15	0.37 ± 0.15	0.37 ± 0.14	0.48 ± 0.23
GSH + GSSG, µmol/mL	22	0.48 ± 0.21	0.43 ± 0.17	0.45 ± 0.17 ^●^	0.79 ± 0.28 ^●^
**Stage III**					
MDA, nmol/mL	8	17.11 ± 11.04	21.66 ± 13.82	14.33 ± 5.38 ^●^	6.73 ± 2.66 ^●^
GST, µmol/mL/min	8	0.41 ± 0.16	0.43 ± 0.17	0.43 ± 0.19	0.48 ± 0.23
GSH + GSSG, µmol/mL	8	0.48 ± 0.16	0.52 ± 0.17	0.56 ± 0.10 ^●^	0.79 ± 0.28 ^●^

GSH + GSSG = total glutathione; GST = glutathione S-transferase; MDA = malondialdehyde; *n* = number of patients. * *p* < 0.017; ^●^ *p* < 0.05.
